# Reversible Hydrogenase Activity Confers Flexibility to Balance Intracellular Redox in *Moorella thermoacetica*

**DOI:** 10.3389/fmicb.2022.897066

**Published:** 2022-05-12

**Authors:** Shunsuke Kobayashi, Junya Kato, Keisuke Wada, Kaisei Takemura, Setsu Kato, Tatsuya Fujii, Yuki Iwasaki, Yoshiteru Aoi, Tomotake Morita, Akinori Matsushika, Katsuji Murakami, Yutaka Nakashimada

**Affiliations:** ^1^Graduate School of Integrated Sciences for Life, Hiroshima University, Higashihiroshima, Japan; ^2^National Institute of Advanced Industrial Science and Technology (AIST), Tsukuba, Japan; ^3^National Institute of Advanced Industrial Science and Technology (AIST), Higashihiroshima, Japan

**Keywords:** acetogen, metabolic engineering, ethanol production, hydrogen inhibition, hydrogen production, redox balance, mixotrophy

## Abstract

Hydrogen (H_2_) converted to reducing equivalents is used by acetogens to fix and metabolize carbon dioxide (CO_2_) to acetate. The utilization of H_2_ enables not only autotrophic growth, but also mixotrophic metabolism in acetogens, enhancing carbon utilization. This feature seems useful, especially when the carbon utilization efficiency of organic carbon sources is lowered by metabolic engineering to produce reduced chemicals, such as ethanol. The potential advantage was tested using engineered strains of *Moorella thermoacetica* that produce ethanol. By adding H_2_ to the fructose-supplied culture, the engineered strains produced increased levels of acetate, and a slight increase in ethanol was observed. The utilization of a knockout strain of the major acetate production pathway, aimed at increasing the carbon flux to ethanol, was unexpectedly hindered by H_2_-mediated growth inhibition in a dose-dependent manner. Metabolomic analysis showed a significant increase in intracellular NADH levels due to H_2_ in the ethanol-producing strain. Higher NADH level was shown to be the cause of growth inhibition because the decrease in NADH level by dimethyl sulfoxide (DMSO) reduction recovered the growth. When H_2_ was not supplemented, the intracellular NADH level was balanced by the reversible electron transfer from NADH oxidation to H_2_ production in the ethanol-producing strain. Therefore, reversible hydrogenase activity confers the ability and flexibility to balance the intracellular redox state of *M. thermoacetica*. Tuning of the redox balance is required in order to benefit from H_2_-supplemented mixotrophy, which was confirmed by engineering to produce acetone.

## Introduction

There is a growing interest in chemical production derived from sources other than fossil fuels. Due to increasing levels of carbon dioxide (CO_2_) in the atmosphere, low-carbon emissions are required to eliminate environmental threats, such as global warming. Technology to capture and utilize CO_2_ as a resource is in progress worldwide, and bioprocessing of renewable feedstocks is one promising candidate. However, economic cost is a bottleneck in bioprocessing applications of bulk chemicals. A means to reduce the cost is to maximize carbon conversion of feedstock to the product.

Acetogens are a group of microorganisms capable of autotrophic growth on CO_2_ and hydrogen (H_2_) and are thus promising chassis for utilizing CO_2_ by bioprocesses (Ljungdhal, 1986; Wood, 1991; Drake, 1994; Drake et al., 2008). The main product is acetate, but some acetogens produce other valuable chemicals, such as ethanol. These by-products can be utilized for industrial production from waste materials, such as off-gas from steel mills. This process, called gas fermentation, has attracted worldwide attention (Bengelsdorf et al., 2016; Liew et al., 2016; Bengelsdorf and Dürre, 2017; Teixeira et al., 2018; Omar et al., 2019; Jin et al., 2020; Kopke and Simpson, 2020; Bourgade et al., 2021; Fackler et al., 2021). On the other hand, acetogens are also capable of heterotrophic growth on various carbohydrate substrates and are good candidates for bioconversion of biomass to useful chemicals. Utilization of acetogens is especially effective for carbon utilization because processing by acetogens emits much less CO_2_ due to the nature of their CO_2_ fixation pathway. When acetogens metabolize hexose to acetate, two molecules of CO_2_ are produced, then reassimilated into the CO_2_ fixation pathway by utilizing reducing equivalents from glycolysis. Therefore, acetogens can theoretically convert one hexose molecule to three acetate molecules (Fontaine et al., 1942; Schuchmann and Müller, 2014, 2016).

Autotrophic and heterotrophic metabolism can be combined for mixotrophic growth, which enables the enhancement of carbon utilization and conversion of extra CO_2_ using H_2_ as the source of reducing power (Fast et al., 2015; Maru et al., 2018). Mixotrophy is a general trait of acetogens and is effective in fermentation, especially for products that are more reduced than acetate. A previous report succeeded in increasing overall metabolite yields by supplying H_2_ to sugar-based cultures of *Clostridium ljungdahlii* (Jones et al., 2016). In this case, a shift in the metabolite profile was observed by providing H_2_, with ethanol as the primary metabolite, over less-reduced products. H_2_ supply for the industrial applications of mixotrophic fermentation would be supported by the development of technology to provide CO_2_-free H_2_ using renewable energy-based approaches, such as water splitting, biomass gasification, and ammonia reforming (Hosseini and Wahid, 2016; Aryal et al., 2018). Thus, together with this technology development to provide H_2_, mixotrophic fermentation would contribute to the low-carbon emitting and economically feasible bioprocesses.

In addition to natural by-products, acetogens can also be engineered to produce chemicals other than acetate. Genetic engineering of acetogens is challenging because of their genetic barrier, such as restriction–modification systems and physical barriers by gram-positive cell walls; however, development of engineering tools has substantially improved the efficiency of engineering acetogens (Minton et al., 2016; Jin et al., 2020; Bourgade et al., 2021). It is also possible to apply metabolic engineering for pathway optimization to enhance the production of target metabolites. Metabolic engineering has begun to highlight the potential of acetogens for chemical production from CO_2_.

*Moorella thermoacetica* is a thermophilic acetogen (Drake and Daniel, 2004; Pierce et al., 2008). Due to its thermophilic nature, *M. thermoacetica* can be used to establish an advantageous bioprocess for the recovery of products, especially volatile chemicals (Taylor et al., 2009; Abdel-Banat et al., 2010; Basen and Müller, 2017; Redl et al., 2017). However, *M. thermoacetica* is categorized as a homoacetogen that produces acetate exclusively. Therefore, the metabolic pathway must be modified to produce other chemicals for industrial applications (Iwasaki et al., 2013, 2017; Kita et al., 2013). We previously succeeded in engineering *M. thermoacetica* to produce ethanol and acetone from sugars and syngas, as well as to enhance yields, by adjusting the carbon flux (Rahayu et al., 2017; Kato et al., 2021; Takemura et al., 2021a). Disruption of the major acetate production pathway enables near-exclusive ethanol production from sugars.

In this study, we attempted to apply H_2_-supplemented mixotrophy to enhance ethanol yield. Unexpectedly, we found that H_2_ supplementation inhibited the growth of a high-ethanol-producing strain. Metabolomic analysis revealed that the engineered strain balanced the intracellular redox status by producing H_2_ to oxidize NADH during heterotrophic growth. Reversible hydrogenase activity, which oxidizes H_2_ in the wild-type strain under standard conditions, plays a vital role in the redox maintenance of metabolically engineered strains. It is necessary to avoid this reverse reaction to fulfill H_2_-supplemented-mixotrophic bioproduction.

## Materials and Methods

### Bacterial Strains and Growth Conditions

*Moorella thermoacetica* ATCC 39073 and its derivatives were used in this study (Table 1). Modified ATCC1754 PETC medium comprising 1.0 g of NH_4_Cl, 0.1 g of KCl, 0.2 g of MgSO_4_·7H_2_O, 0.8 g of NaCl, 0.1 g of KH_2_PO_4_, 0.02 g of CaCl_2_·2H_2_O, 2.0 g of NaHCO_3_, 10 ml of trace elements, 10 ml of Wolfe’s vitamin solution (Tanner, 1989), and 1.0 mg of resazurin/L of deionized water was used as the basal medium (Tanner et al., 1993). The pH of the solution was adjusted to 6.9. The medium was prepared anaerobically by boiling and cooling under an N_2_–CO_2_ (80:20) mixed-gas atmosphere. After cooling, the medium was dispensed into 125 ml glass culture vials (serum bottles) under an N_2_–CO_2_ mixed-gas atmosphere. The vials were crimp-sealed and autoclaved.

**Table 1:** Strains used in this study.

Strain name	Relevant characteristics	References
Wild type	ATCC 39073	ATCC
Mt-*aldh*	*pyrF*::*aldh*	Rahayu et al., 2017
Mt-Δ*pduL2*::*aldh*	Δ*pduL2*::*aldh*	Rahayu et al., 2017
Mt-Δ*pduL1*Δ*pduL2*::*aldh*	Δ*pduL1* Δ*pduL2*::*aldh*	Rahayu et al., 2017
pduL2::acetone	Δ*pduL2*::*ctfAB*-*thl*-*adc*	Kato et al., 2021

Before starting the culture, fructose, yeast extract, and L-cysteine·HCl·H_2_O were added to reach final concentrations of 2.0, 1.0, and 1.2 g/l, respectively. The final volume was adjusted to 50 ml with water. To provide H_2_, the headspace pressure in the vials was adjusted to 0.12 MPa by using N_2_–CO_2_ (80:20) mixed-gas. H_2_ gas was then injected at the desired pressure. For example, when 0.01 MPa of H_2_ was tested, the total pressure was adjusted to 0.13 MPa by the H_2_ gas injection. Cells were grown at 55°C with shaking at 180 rpm.

### Analytical Methods

We sampled and analyzed 1 ml of the culture medium at each time point and calculated the dry cell weight using the optical density (OD) at 600 nm [OD_600_; 1 g (dry cell weight)/L = 0.383 OD_600_; Iwasaki et al., 2017]. The culture supernatant was analyzed for the amount of fructose, formate, acetate, ethanol, and acetone using high-performance liquid chromatography (HPLC; LC-2000 Plus HPLC; Jasco, Tokyo, Japan) equipped with a refractive index detector (RI-2031 Plus; Jasco), Shodex RSpak KC-811 column (Showa Denko, Kanagawa, Japan), and Shodex RSpak KC-G guard column (Showa Denko) at 60°C. Ultrapure water containing 0.1% (v/v) phosphoric acid was used as the mobile phase at a flow rate of 0.7 ml/min, and crotonate was used as the internal standard (Miura et al., 2014). The gas composition in the headspace of the culture vials was analyzed using GC-8A gas chromatograph (Shimadzu, Kyoto, Japan) equipped with a thermal conductivity detector and a stainless steel column packed with activated carbon at 70°C. Argon was used as the carrier gas (Miura et al., 2014). The total gas pressure in the headspace was measured using a differential pressure gauge (DMC-104 N11; Okano Works, Tokyo, Japan).

### Metabolome Analysis

Strains were grown to reach the exponential phase between 0.5 and 0.7 OD_600_. The culture was immediately filtered to collect cells equivalent to a total count of 20 OD_600_ (volume [mL] × OD_600_ ≈ 20). Filtration was performed using hydrophilic PTFE, 1 μm pore size, and a 90-mm-diameter filter disk (Omnipore; Merck KGaA, Darmstadt, Germany). Harvested cells were immediately immersed in pre-chilled methanol containing 100 μM ribitol and 100 μM (+)-10-camphorsulfonate to quench the metabolic activity. This procedure was quickly performed, within 45 s after opening culture vials, to avoid metabolites from artifacts, such as those caused by oxygenation, degradation, and other modifications. Subsequently, intracellular metabolites were extracted using the chloroform–water–methanol method (Bolten et al., 2007). The supernatant was then concentrated using a centrifugal concentrator (CC-105; Tomy, Tokyo, Japan). According to a previous study, pre-treatment and analysis of the dried samples were performed (Wada et al., 2022).

## Results

### H_2_ Supplementation Increases Carbon Utilization in Mixotrophic Growth by Producing Acetate, Not Ethanol, in Engineered Strains

**Figure 1:**
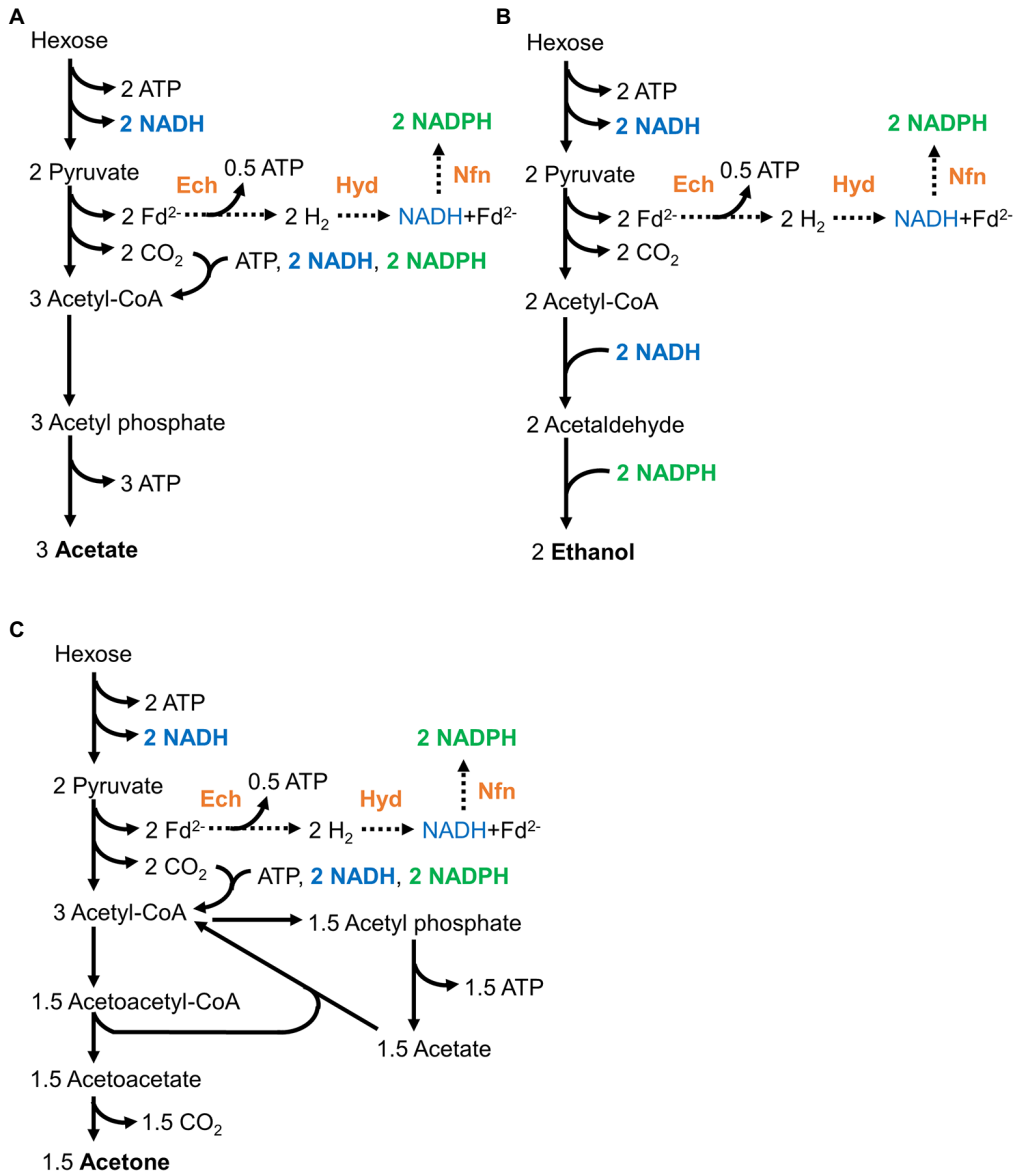
Redox-balanced pathways for acetogenesis **(A)**, ethanol production **(B)**, and acetone production **(C)** from hexose in wild-type and engineered strains for ethanol production. NADH (shown in blue) and the reduced forms of ferredoxin are produced during glycolysis and the conversion of pyruvate to acetyl-CoA. The reduced ferredoxin is converted to NADPH (shown in green) *via* hydrogenases and electron-bifurcating enzymes, that is, the Ech complex, HydABC, and NfnAB complexes (shown in orange).

*Moorella thermoacetica* can convert one hexose molecule to three acetate molecules in theory (Figure 1A; Fontaine et al., 1942; Schuchmann and Müller, 2014, 2016). The engineered strains were designed to produce ethanol from acetyl-CoA in two steps (reducing reactions; Rahayu et al., 2017; Table 1). The reducing equivalents provided by glycolysis were assumed to be properly consumed (Figures 1A,B). In a model, the Ech complex, HydABC complex, and NfnAB complex would convert the reducing equivalents to NADH and NADPH. These NADH and NADPH would be consumed by the Wood–Ljungdahl pathway to convert CO_2_ to acetate in the wild-type strain, whereas reduction of acetyl-CoA would consume the NADH and NADPH in the ethanol-producing strains. Therefore, the redox conditions in the engineered strains producing ethanol should be balanced. In fact, we previously observed that all engineered strains (Table 1) grew and produced ethanol on hexose sugars. However, one molecule of CO_2_ is released to produce one molecule of ethanol because of the requirement for extra reducing equivalents (approximately 33% of the carbon is released from hexose sugars in theory). Therefore we supplied H_2_ to increase carbon utilization by mixotrophy.

**Figure 2:**
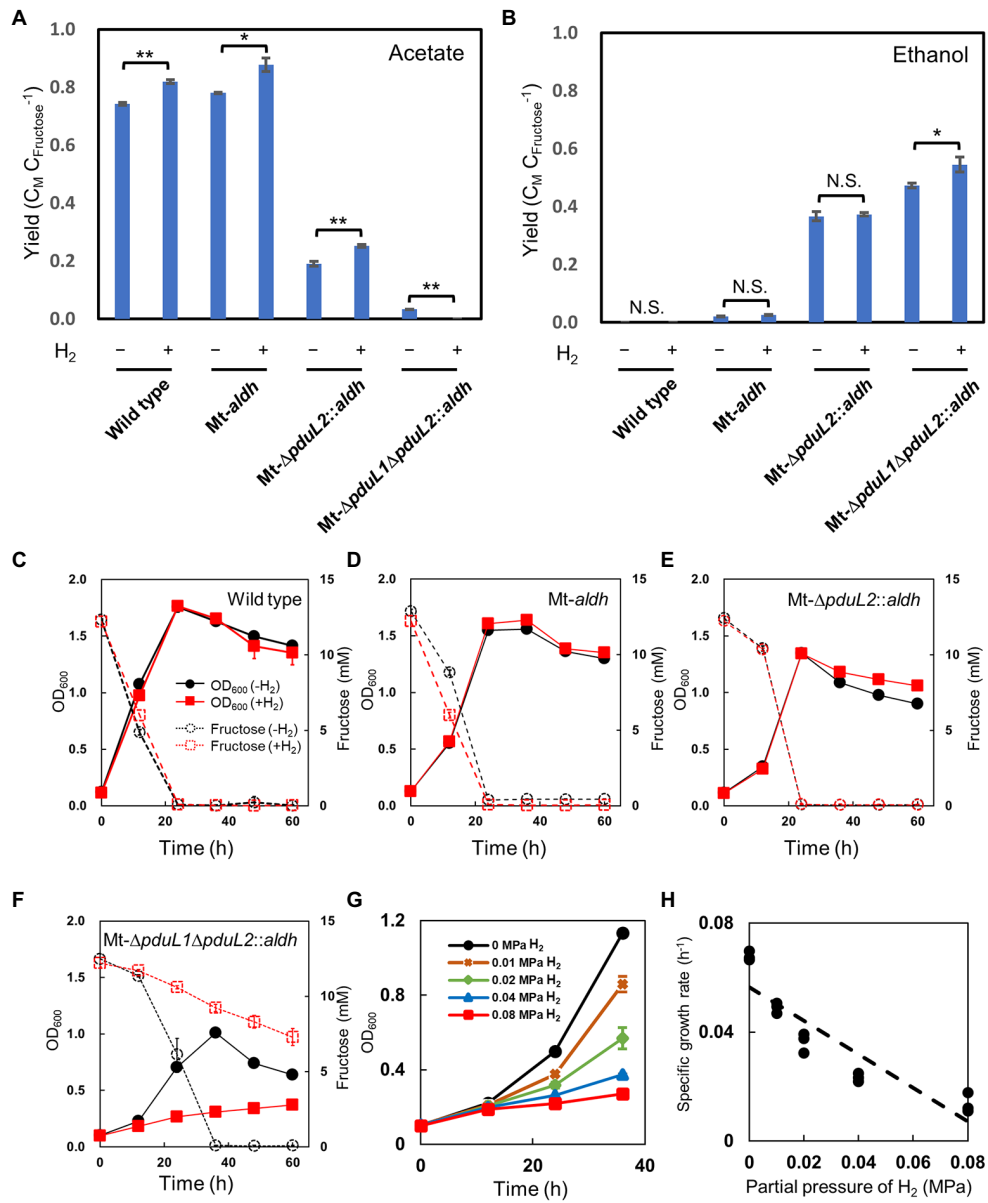
Effect of H_2_ supplementation on the wild-type strain and the engineered strains for ethanol production in mixotrophic conditions. **(A,B)** Product profiles shown as carbon molar yields (C_M_ C_Fructose_^−1^; C_Metabolite_ C_Fructose_^−1^). Fructose was used as the carbohydrate substrate. The amount of acetate and ethanol production was compared with and without supplementation of H_2_ (0.08 MPa partial pressure, 40% in the headspace) after 60 h when all the supplied fructose was consumed in the no H_2_-supplied condition. *t*-test was performed to evaluate the significance. N.S., not significant; value of ^*^p<0.05; value of ^**^p<0.01. **(C-** **F)** Profiles for cell growth and fructose consumption. The culture was same as in **(A,B)**. Cell density was shown as OD_600_ (optical density at 600 nm) by solid lines. Fructose concentration was shown by dotted lines. Black, no H_2_-supplied condition; red, H_2_-supplied condition. **(G)** Growth profiles of the Mt-Δ*pduL1*Δ*pduL2*::*aldh* strain with various H_2_ partial pressures. The profiles of the exponential phase were compared. The standard deviation (SD) of three biological replicates is shown by error bars. Some error bars are smaller than the symbols. **(H)** Specific growth rate plotted against H_2_ partial pressure. All data shown were obtained in the culture for **(G)**.

We used a culture containing fructose as the carbohydrate substrate. H_2_ was added to the headspace of the culture vial at a partial pressure of 0.08 MPa (equivalent to 40% of the gas phase). The gas phase also contained CO_2_, and therefore extra CO_2_ could be incorporated in addition to the released CO_2_. Of the injected gas, CO_2_ was 12% of the total, and the medium contained NaHCO_3_ to supply CO_2_. First, we tested the effect of H_2_ on the wild-type strain. Acetate was produced as the end product and the carbon molar yield improved from 0.74 to 0.82, as expected (Figures 2A,B). The optical density increased similarly while fructose was consumed, and decreased after the complete consumption of fructose in both conditions, indicating no significant effect on the growth (Figure 2C). We then tested an ethanol-producing strain, Mt-*aldh*, in which the *aldh* gene encoding aldehyde dehydrogenase was expressed by a constitutive promoter. The main product was acetate, accompanied by a small amount of ethanol. This trend was similar in the H_2_-supplied culture, and yield improvement was only observed for acetate production from 0.78 to 0.88 (Figure 2A). The change in ethanol production was not significant (0.02 and 0.03; Figure 2B). No significant effect on the growth was observed (Figure 2D). Although we expected to enhance the yield of reduced products, we reasoned that the abundant activity of the acetate production pathway in the Mt-*aldh* strain decreased the effect of H_2_. We then tested another ethanol-producing strain, Mt-Δ*pduL2*::*aldh*, which showed less acetate production due to deletion of one of the two genes (*pduL2*) encoding phosphoacetyl transferase in the acetate production pathway. Despite the dominant production of ethanol over acetate, product yield enhancement was observed with only acetate from 0.19 to 0.25 (Figures 2A,B). The carbon molar yield for ethanol did not change (0.37). The rest was released as CO_2_. The strain grew similarly in both conditions (Figure 2E). The effect of H_2_ was in contrast to the results of a previous study, in which the supplementation of reducing power with H_2_ was reflected in the production of more-reduced chemicals (Jones et al., 2016).

### H_2_ Supplementation Causes Growth Inhibition of the Engineered Strain, Which Exclusively Produces Ethanol

We tested the Mt-Δ*pduL1*Δ*pduL2*::*aldh* strain, which produces almost exclusively ethanol, because of the complete knockout of both two genes (*pduL1* and *pduL2*) encoding phosphoacetyl transferase. In this case, we observed an enhancement in the ethanol yield. The carbon molar yield for ethanol was increased from 0.47 to 0.54 (Figure 2B). Approximately 50% of the carbon from fructose was released as CO_2_ in the absence of H_2_, and H_2_ supplementation supported capture and conversion of CO_2_. However, the supplemented fructose was not completely consumed in the H_2_-supplied condition after 60 h of cultivation, whereas the same strain in the H_2_-unsupplied condition, or the other strains in both conditions, consumed fructose completely (Figures 2C–F). Only 40.2% of the supplemented fructose was consumed in the H_2_-supplied condition, and the growth was significantly reduced in a correlated manner (Figure 2F). The volumetric amount of ethanol was also significantly less in the H_2_-supplied condition, showing only 45.5% against the no-H_2_ condition (17.6 mM in the absence of H_2_ and 8.0 mM in the presence of H_2_), reflecting the amount of consumed fructose. Therefore, although the Mt-Δ*pduL1*Δ*pduL2*::*aldh* strain showed increased carbon molar yield for ethanol under H_2_-supplemented mixotrophic conditions, growth inhibition emerged as an unexpected bottleneck.

We also tested the addition of different amounts of H_2_ to the Mt-Δ*pduL1*Δ*pduL2*::*aldh* strain. In addition to the condition of partial pressure 0.08 MPa, we tested 0.04, 0.02, and 0.01 MPa, because the pressure of H_2_ is correlated with dissolved H_2_ in the culture medium. All cases with H_2_ at any concentration showed growth inhibition effects. Interestingly, the effect of growth inhibition was dose-dependent, showing more potent inhibition by a higher concentration of H_2_ in the culture medium, rather than by a certain threshold. This tendency was clear when the growth rate was plotted against the H_2_ pressure, showing a linear correlation (Figure 2H).

### Metabolome Analysis Identifies Specific and Significant Enhancement of Intracellular NADH Level by H_2_ Supplementation in the Ethanol-Producing Strain

**Figure 3:**
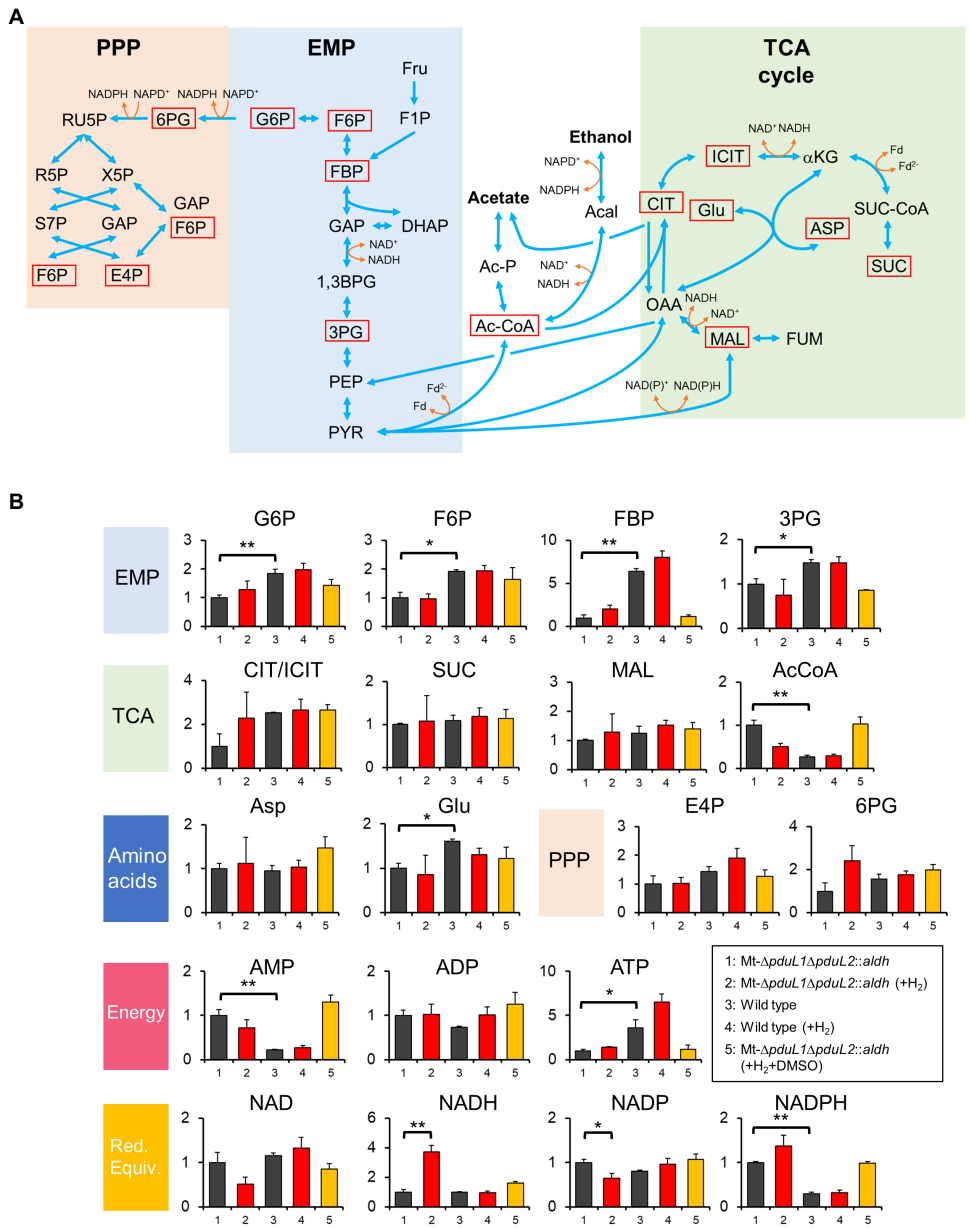
Metabolome analysis of the Mt-Δ*pduL1*Δ*pduL2*::*aldh* strain and the wild-type strain with and without H_2_ supplementation. **(A)** Metabolic pathways for the *Moorella thermoacetica* strains based on KEGG database (https://www.genome.jp/kegg-bin/show_organism?org=mta) and a previous study on a metabolic model (Islam et al., 2015). Metabolites quantified except for cofactors are shown by red squares. EMP, Embden–Meyerhof–Parnas pathway; PPP, pentose phosphate pathway; Fru, fructose; F1P, fructose 1-phosphate; G6P, glucose-6-phosphate; F6P, fructose 6-phosphate; FBP, fructose-1,6-bisphosphate; GAP, glyceraldehyde 3-phosphate; DHAP, dihydroxyacetone phosphate; 1,3BPG, 1,3-bisphosphoglyceric acid; 3PG, 3-phosphoglyceric acid; PEP, phosphoenolpyruvic acid; PYR, pyruvate; X5P, xylulose 5-phosphate; RU5P, ribulose 5-phosphate; R5P, ribose 5-phosphate; S7P, sedoheptulose 7-phosphate; E4P, erythrose 4-phosphate; Ac-CoA, Acetyl-CoA; Ac-P, acetyl phosphate; Acal, acetaldehyde; CIT, citrate; ICIT, isocitrate; αKG, alpha-ketoglutarate; SUC-CoA, succinyl-CoA; SUC, succinate; Glu, glutamate; OAA, oxaloacetic acid; MAL, malate; FUM, fumarate; ASP, aspartate; GAP, glyceraldehyde 3-phosphate. **(B)** Relative concentrations of intracellular metabolites for the Mt-Δ*pduL1*Δ*pduL2*::*aldh* strain and the wild-type strain in the absence (black) and presence of H_2_ (0.02 MPa, red) on fructose as the carbohydrate substrate. The experiment with DMSO supplementation in addition to H_2_ is shown in orange. All the cell samples were collected at around 0.6 of OD_600_. DMSO was added at 0.4 of OD_600_. For each sample, values were normalized to the Mt-Δ*pduL1*Δ*pduL2*::*aldh* strain without H_2_ supplementation. The vertical axis represents a unitless ratio of metabolite concentrations. Error bars represent the SD of at least two biological replicates. Red. Equiv. represents reducing equivalents. *t*-test was performed to evaluate the significance between data sets 1 and 2, data sets 3 and 4, and data sets 1 and 3. Significant differences are shown. value of ^*^p<0.05; value of ^**^p<0.01.

To investigate the H_2_-dependent growth inhibition mechanism of the ethanol-producing strain, we assessed intracellular metabolism by metabolome analysis. We used H_2_ at 0.02 MPa, because a high dose of H_2_ (such as 0.08 MPa) inhibited the growth almost completely, and hence might have significant effects on multiple metabolic pathways. We sampled the cells from the exponential phase and analyzed intracellular metabolites using GC–MS and LC–MS following the sample preparation method we developed. We succeeded in quantifying 19 intracellular metabolites, including seven cofactors (Figures 3A,B).

We then compared the Mt-Δ*pduL1*Δ*pduL2*::*aldh* strain and the wild-type strain, with or without H_2_ supplementation. Among all analyzed metabolites, NADH levels were striking in the Mt-Δ*pduL1*Δ*pduL2*::*aldh* strain under H_2_-supplied conditions. The NADH level increased by approximately four times compared to that in the no H_2_ condition, whereas H_2_ supplementation did not affect the NADH level in the wild-type strain. There was no such significant difference specific to H_2_ supplementation in the other metabolites in the Mt-Δ*pduL1*Δ*pduL2*::*aldh* strain or metabolites in the wild-type strain (Figure 3B).

When the overall metabolite profiles were compared in the Mt-Δ*pduL1*Δ*pduL2*::*aldh* and wild-type strains in the no H_2_ condition, the levels of glucose-6-phosphate, fructose-6-phosphate, fructose-1,6-bisphosphate, 3-phosphoglyceric acid, glutamate, and ATP were lower, and the levels of acetyl-CoA, AMP, and NADPH were higher in the Mt-Δ*pduL1*Δ*pduL2*::*aldh* strain. The ATP level was lowered in the Mt-Δ*pduL1*Δ*pduL2*::*aldh* due to the knockout of acetate production coupled with substrate-level phosphorylation, but the ATP level was enough to maintain the growth (Figure 2F). In contrast, the AMP level was higher in the Mt-Δ*pduL1*Δ*pduL2*::*aldh*, and this may be related with the change of ATP level. The higher level of acetyl-CoA probably reflects a difference of conversion rate of acetyl-CoA to ethanol and acetate. The higher level of NADPH in the Mt-Δ*pduL1*Δ*pduL2*::*aldh* strain suggests that the redox balance in this strain was altered by metabolic engineering. Metabolome analysis indicated that the Mt-Δ*pduL1*Δ*pduL2*::*aldh* strain suffered redox imbalances due to both metabolic engineering and H_2_ supplementation.

### Hydrogen Production by the Ethanol-Producing Strain

**Figure 4:**
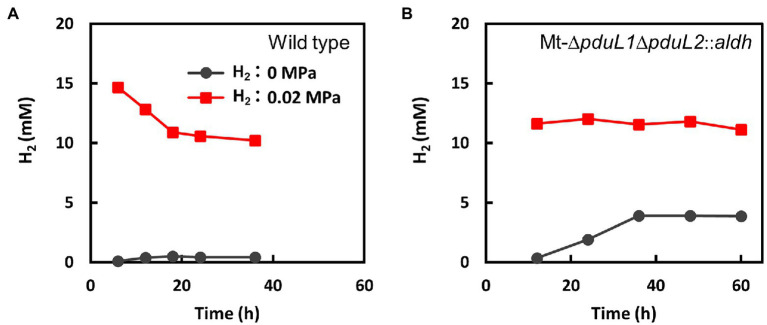
Monitoring the consumption and evolution of H_2_ by the wild-type strain **(A)** and the Mt-Δ*pduL1*Δ*pduL2*::*aldh* strain **(B)**. The total amount of H_2_ was divided by the volume of the culture medium. Black, no H_2_-supplied condition; red, H_2_-supplied condition (0.02 MPa). Error bars, which are smaller than symbols, are the SD of three biological replicates.

Metabolomic analysis suggested a strong relationship between growth inhibition by H_2_ and increased levels of intracellular NADH. In *M. thermoacetica*, NAD^+^ is reduced to NADH by the electron-bifurcating hydrogenase HydABC complex (Wang et al., 2013). The HydABC complex reduces NAD^+^ and ferredoxin using electrons from H_2_. This reaction is reversible and produces H_2_ from NADH and reduced ferredoxin *in vitro*. Therefore, we measured the amount of H_2_ in the headspace of culture vials (Figure 4). The amount of H_2_ in the headspace was traced in conditions with or without H_2_ supplementation, and the wild-type strain and the Mt-Δ*pduL1*Δ*pduL2*::*aldh* strain were compared. H_2_ was supplied at 0.02 MPa of a partial pressure, in addition to fructose as a carbohydrate substrate, which was the same condition for our metabolome analysis. There was almost no H_2_ production by the wild-type strain, and the supplied H_2_ was consumed over time (Figure 4A). In contrast, the Mt-Δ*pduL1*Δ*pduL2*::*aldh* strain apparently did not consume H_2_ under the same conditions (Figure 4B). Moreover, when H_2_ was not supplied, the H_2_ level increased in the culture vial of the Mt-Δ*pduL1*Δ*pduL2*::*aldh* strain, in contrast to that in the wild-type strain. H_2_ production is usually attributed to the disposal of excess electrons from metabolism. In this case, this was most likely due to the increased level of NADH. However, H_2_ formation would require a sufficiently low level of H_2_ as the product. Therefore, the Mt-Δ*pduL1*Δ*pduL2*::*aldh* strain would have produced H_2_ for the clearance of the excess electrons from catabolizing fructose, and H_2_ supplementation would inhibit the H_2_ production, causing the growth inhibition due to the redox imbalance.

### NADH Consumption by DMSO Reduction Prevents the Growth Inhibition by H_2_

**Figure 5:**
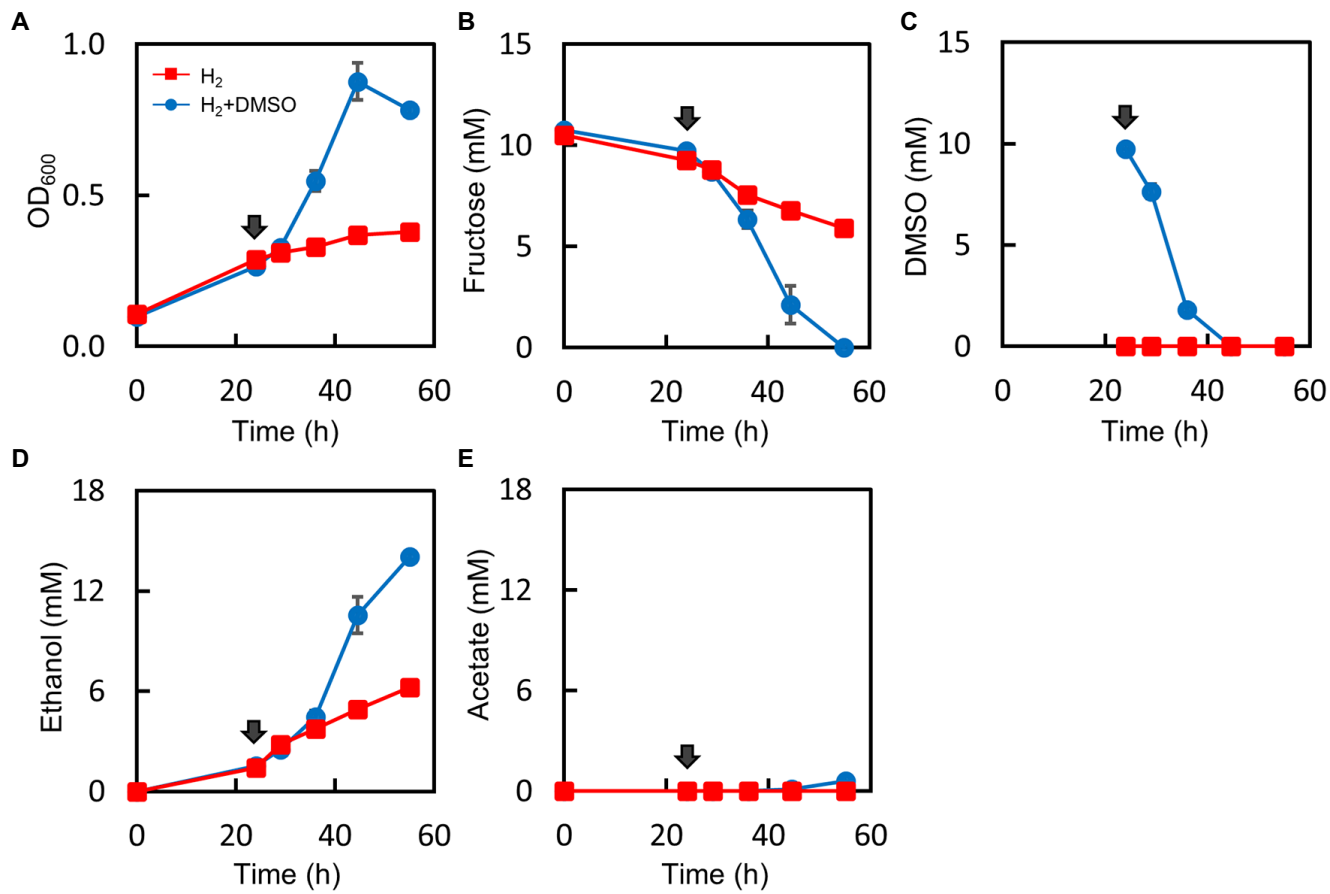
Effect of DMSO supplementation on the culture profile in the H_2_-supplemented mixotrophic condition of the Mt-Δ*pduL1*Δ*pduL2*::*aldh* strain. H_2_ (0.08 MPa) was supplied to the fructose-supplemented culture, and DMSO (10 mM) was added to one group of the culture medium after 24 h (shown by arrows). The DMSO-supplied condition is shown in blue, whereas the no-DMSO condition is shown in red. Each graph shows the culture profiles for **(A)**, OD_600_; **(B)**, Fructose concentration; **(C)**, DMSO concentration; **(D)**, Ethanol production; **(E)**, Acetate production. Error bars represent the SD of three biological replicates.

The results of the H_2_ measurement strongly indicated that the Mt-Δ*pduL1*Δ*pduL2*::*aldh* strain produced H_2_ using excess reducing equivalents and balanced the intracellular redox. Our metabolome analysis showed imbalanced redox in the Mt-Δ*pduL1*Δ*pduL2*::*aldh* strain, manifested as an increased level of intracellular NADH. If a high level of intracellular NADH is the direct cause of growth inhibition, the Mt-Δ*pduL1*Δ*pduL2*::*aldh* strain should recover its growth in the presence of H_2_ by lowering the intracellular NADH level. *M. thermoacetica* uses dimethyl sulfoxide (DMSO) as an electron acceptor, and the reported cases of bacterial DMSO reduction are NADH-dependent reactions (Zinder and Brock, 1978; De Bont et al., 1981; Drake and Daniel, 2004; Takemura et al., 2021b; Rosenbaum et al., 2022). We attempted to oxidize intracellular NADH *via* DMSO reduction by supplementing the culture medium with DMSO. We set up a culture of the Mt-Δ*pduL1*Δ*pduL2*::*aldh* strain with H_2_ supplied at 0.08 MPa of partial pressure in addition to fructose, which showed strong growth inhibition (Figures 2F–H). At 24 h, the culture was supplied with 10 mM DMSO. The Mt-Δ*pduL1*Δ*pduL2*::*aldh* strain showed very slow growth with H_2_ supplementation, but the growth rate dramatically increased upon DMSO supplementation (Figure 5A). The fructose supplied was completely consumed after 55 h (Figure 5B). The supplied DMSO was readily consumed within 20 h after the DMSO addition, consistent with the recovery of growth and fructose consumption (Figure 5C). The addition of DMSO also improved total ethanol production, whereas acetate production remained minor (Figures 5D,E).

We analyzed the effect of DMSO on the intracellular metabolome in the presence of H_2_ (Figure 3B). We used the same culture conditions as in the metabolome analysis (H_2_ partial pressure = 0.02 MPa), except for DMSO supplementation, which was provided when the cells entered the exponential phase. As expected, the intracellular level of NADH was lower than that in the H_2_ condition. Therefore, growth recovery correlated with intracellular NADH levels.

### H_2_ Enhances Target Metabolite Production by a Metabolically Engineered Strain With Balanced Redox

We found that the ethanol production pathway designed to balance the redox reaction requires tuning the imbalanced redox by producing H_2_. This means that our ethanol-producing strains need to be re-engineered to balance the redox reaction to benefit from H_2_-supplemented mixotrophy. However, it is possible that artificial modifications in the genome can affect metabolic activities in an unpredictable manner. We previously succeeded in engineering *M. thermoacetica* to produce acetone (Kato et al., 2021; Table 1). The acetone synthesis pathway does not require any oxidoreductases to convert acetyl-CoA to acetone, which has the same redox balance as that of the native acetate pathway (Figure 1C). Therefore, acetone production has completely the same redox balance as acetate production, and the redox balance should not be affected. On the other hand, the pduL2::acetone strain has the same elements of genetic modification as an ethanol-producing strain, Mt-Δ*pduL2*::*aldh*, using a *pyrF* marker for selection and a constitutive G3PD (glyceraldehyde 3-phosphate dehydrogenase) promoter to express *aldh*, disrupting the acetate pathway. Therefore, we examined whether the introduction of an oxidoreductase affected the redox balance by testing the H_2_-supplemented mixotrophic acetone production of the pduL2::acetone strain.

**Figure 6:**
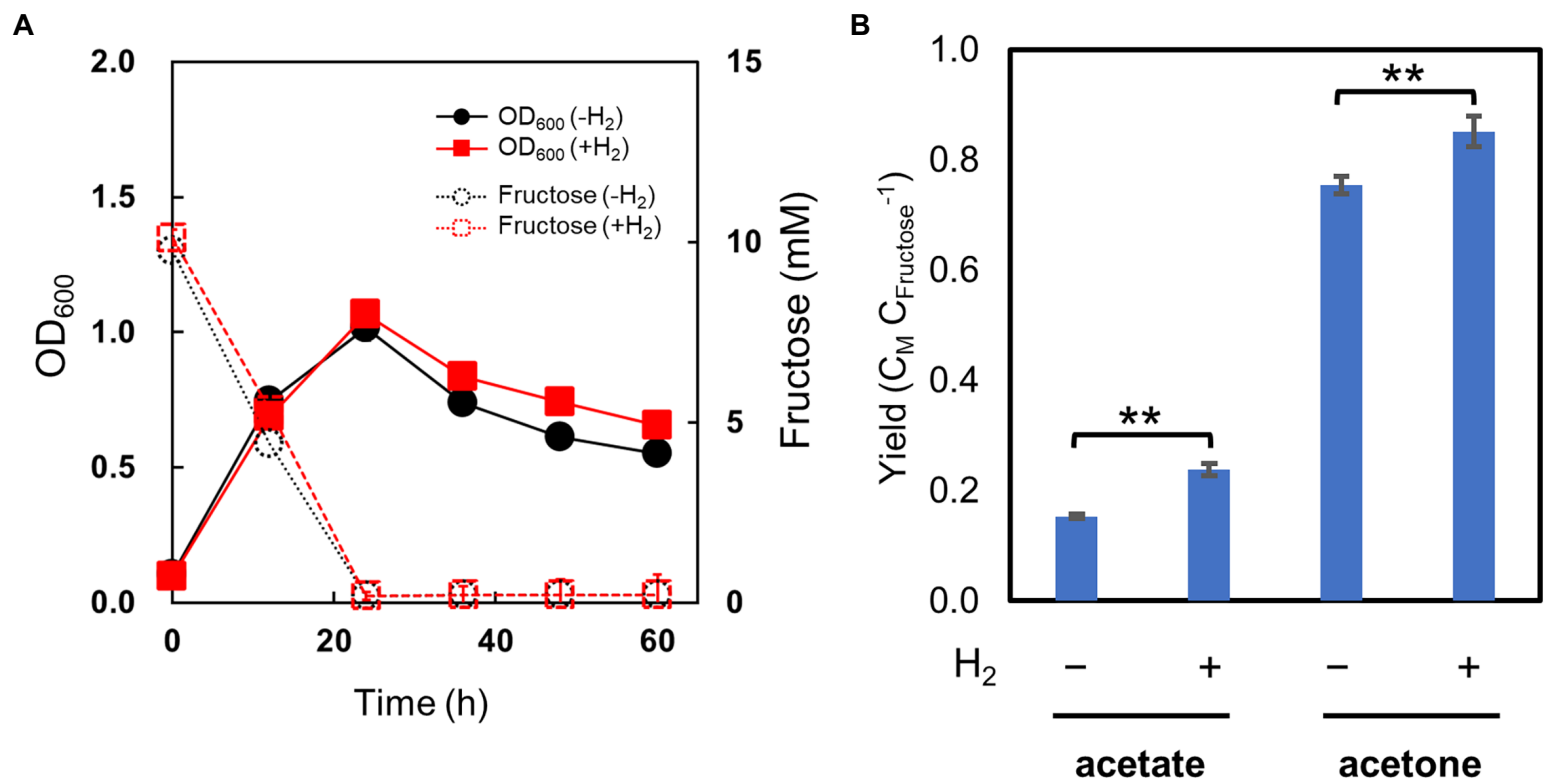
Culture profiles of the metabolically engineered strain of *Moorella thermoacetica* for acetone production in the H_2_-supplemented mixotrophic condition. **(A)** shows cell growth (solid lines) and fructose consumption (dotted lines) in the absence (black) and presence (red) of H_2_. **(B)** shows carbon molar yields for acetate and acetone production. Error bars represent the SD of three biological replicates, some of which are smaller than symbols.

We set up cultures of the pduL2::acetone strain with fructose as the carbohydrate substrate and tested the effect of H_2_ supplementation, as was performed for ethanol-producing strains. H_2_ was supplied at 0.08 MPa of partial pressure, the highest dose used for the ethanol-producing strains. The pduL2::acetone strain grew in the presence of H_2_ in the same manner as in its absence (Figure 6A). The optical density increased while fructose was consumed, and decreased after the complete consumption of fructose in both cases. Furthermore, the produced acetone and acetate increased with H_2_ supplementation (Figure 6B), in contrast to ethanol production. Acetone production was enhanced by 13%, and the total carbon molar yield (the sum of acetate and acetone) was greater than one, indicating that extra CO_2_ was converted to these metabolites. CO_2_ was externally provided in the headspace gas and from NaHCO_3_ in the medium, in addition to CO_2_ released from metabolism. Engineering to introduce a non-reductive pathway did not erase the effect of mixotrophy. Therefore, we concluded that introducing oxidoreductase reactions to convert acetyl-CoA to ethanol caused H_2_ production by extra electrons and H_2_ inhibition.

## Discussion

Metabolic engineering to divert acetate to more reduced chemicals lowers carbon utilization and releases more CO_2_ in acetogens due to the loss of the reducing power to fix and convert CO_2_ for oxidoreductase reactions. One strategy for overcoming this issue is H_2_-supplemented mixotrophy. However, our engineered strains, Mt-*aldh* and Mt-Δ*pduL2*::*aldh*, produced an increased amount of acetate instead of ethanol. Growth inhibition was observed in the Mt-Δ*pduL1*Δ*pduL2*::*aldh* strain. Therefore, the strategy for enhancing carbon utilization by H_2_ is not effective for metabolically engineered strains in terms of ethanol production.

Growth inhibition due to H_2_ supplementation has been reported in several studies. For example, one classical case involves *Clostridium cellobioparum*, an H_2_ producer. When H_2_ was added to the culture, *C. cellobioparum* growth was inhibited in an H_2_ dose-dependent manner (Chung, 1976). This trend was similar to that of our ethanol-producing strain, Mt-Δ*pduL1*Δ*pduL2*::*aldh*. The study also reported that the removal of H_2_ recovered the growth using a catalyst, gassing out, or co-culture with methanogenic microorganisms. *C. cellobioparum* is a resident of the bovine rumen, living together with methanogens; therefore, the H_2_ level remains low and does not affect their growth *in situ* (Hungate, 1967; Hungate et al., 1970). Another example was observed in the case of a thermophilic microorganism for H_2_ bioproduction, *Caldicellulosiruptor saccharolyticus* (Willquist et al., 2011). A high level of H_2_ produced by *C. saccharolyticus* inhibits its own growth, demanding continuous stripping of the produced H_2_ from fermentation. Interestingly, the desired by-product for high H_2_ yields is acetate, because more-reduced products, such as ethanol, drain electrons from H_2_ production. When H_2_ levels increase, *C. saccharolyticus* produces lactate and ethanol instead of H_2_ to oxidize NADH and maintain the NADH/NAD ratio. In contrast, our metabolically engineered ethanol-producing strain of *M. thermoacetica* produced H_2_ instead of ethanol to oxidize NADH. *M. thermoacetica* has been reported to evolve H_2_ under certain conditions, such as in a CO-supplemented culture with glucose (Martin et al., 1983). The intracellular activity level of hydrogenases is significantly enhanced by CO, but not by other gas phases, including H_2_ (Kellum and Drake, 1984). *M. thermoacetica* does not evolve H_2_ in a standard culture under heterotrophic conditions, as seen in our experiment.

Metabolomic analysis revealed that the Mt-Δ*pduL1*Δ*pduL2*::*aldh* strain could maintain NADH levels in the absence of H_2_. This was due to H_2_ production for NADH oxidation, and might also be due to NADPH production using the NfnAB complex. The NfnAB complex transfers electrons from reduced ferredoxin and NADH to NADP^+^ (Huang et al., 2012). However, because the Mt-Δ*pduL1*Δ*pduL2*::*aldh* strain possesses a high NADPH level, the conversion of reduced ferredoxin and NADH to NADPH would be inhibited or difficult. It is unclear why the basal level of NADPH in the Mt-Δ*pduL1*Δ*pduL2*::*aldh* strain was higher than that in the wild-type strain. One possibility is the slow conversion of acetyl-CoA to ethanol, because metabolome analysis showed that the intracellular level of acetyl-CoA was higher in the Mt-Δ*pduL1*Δ*pduL2*::*aldh* strain (Figure 3B). The reaction speed may have limited the consumption of cofactors NADH and NADPH, and the cofactors of the reduced forms accumulated. Although NADH production can be balanced by H_2_ formation, NADPH production cannot be balanced. The Mt-Δ*pduL1*Δ*pduL2*::*aldh* strain could grow and produce ethanol in non-H_2_-supplemented conditions, but NADH could not be balanced upon H_2_ supplementation due to the blockage of H_2_ production. Another possibility is unregulated NADPH production by the pentose phosphate pathway caused by unknown mechanisms due to metabolic engineering. Although a high level of NADPH remained, even in the presence of DMSO to consume NADH under H_2_-supplemented conditions (Figure 3B), the NADPH level did not inhibit growth. Growth inhibition was caused by high NADH levels. We assume that the target reaction influenced by NADH level is glyceraldehyde 3-phosphate dehydrogenase, because this is NADH-dependent (Thauer, 1972; Huang et al., 2012). In studies on ethanol tolerance, glyceraldehyde 3-phosphate dehydrogenase was found to be inhibited by high levels of NADH in *Clostridium thermocellum* (Tian et al., 2017).

Finally, we confirmed that the increased acetate formation, but not ethanol formation, was due to the redox balance itself in the Mt-Δ*pduL2*::*aldh* strain. An engineered strain for acetone production produced higher levels of acetate and acetone with H_2_ supplementation. Therefore, our strategy for gene manipulation itself did not affect redox balance, and the usefulness of H_2_-supplemented mixotrophy was confirmed. The introduction of oxidoreductases affected the redox balance in ethanol-producing strains; hence, H_2_ supplementation only enhanced acetate or inhibited growth. Although the metabolic pathway was designed to be redox-balanced by choosing oxidoreductases with appropriate cofactors (Figure 1), fine-tuning and a different design for properly balanced redox is required to derive benefits from H_2_-supplemented mixotrophy for ethanol production. In contrast, if H_2_ production is the aim of engineering, a metabolic design to increase the intracellular NADH level is one strategy to exploit the hydrogenase reaction.

## Data Availability Statement

The original contributions presented in the study are included in the article/supplementary material, and further inquiries can be directed to the corresponding author.

## Author Contributions

JK and YN conceived and designed the experiments. ShK, JK, and KW performed the experiments. ShK, JK, KW, KT, SeK, TF, YI, YA, TM, AM, and KM analyzed the data. ShK, JK, and KW visualized the data. JK prepared the manuscript. YN supervised the project. All authors have contributed to the manuscript and approved the submitted version.

## Funding

Part of this work was supported by JSPS KAKENHI Grant Number 18 K04853 and JST-Mirai Program Grant Number JPMJMI18E5, Japan.

## Conflict of Interest

The authors declare that the research was conducted in the absence of any commercial or financial relationships that could be construed as potential conflicts of interest.

## Publisher’s Note

All claims expressed in this article are solely those of the authors and do not necessarily represent those of their affiliated organizations, or those of the publisher, the editors and the reviewers. Any product that may be evaluated in this article, or claim that may be made by its manufacturer, is not guaranteed or endorsed by the publisher.
